# Multifunctional Silica-Based Nanoparticles with Controlled Release of Organotin Metallodrug for Targeted Theranosis of Breast Cancer

**DOI:** 10.3390/cancers12010187

**Published:** 2020-01-12

**Authors:** Karina Ovejero Paredes, Diana Díaz-García, Victoria García-Almodóvar, Laura Lozano Chamizo, Marzia Marciello, Miguel Díaz-Sánchez, Sanjiv Prashar, Santiago Gómez-Ruiz, Marco Filice

**Affiliations:** 1 Nanobiotechnology for Life Sciences Group, Department of Chemistry in Pharmaceutical Sciences, Faculty of Pharmacy, Universidad Complutense de Madrid (UCM), Plaza Ramón y Cajal s/n, E-28040 Madrid, Spain; kovejero@ucm.es (K.O.P.); v.garciaalm@alumnos.urjc.es (V.G.-A.); laurloza@ucm.es (L.L.C.); marmarci@ucm.es (M.M.); 2 Microscopy and Dynamic Imaging Unit, Fundación Centro Nacional de Investigaciones Cardiovasculares Carlos III (CNIC), Calle Melchor Fernandez Almagro 3, E-28029 Madrid, Spain; 3 COMET-NANO Group. Department of Biology and Geology, Physics and Inorganic Chemistry, ESCET, Universidad Rey Juan Carlos, Calle Tulipán s/n, E-28933 Móstoles (Madrid), Spain; diana.diaz@urjc.es (D.D.-G.); miguel.diaz@urjc.es (M.D.-S.); sanjiv.prashar@urjc.es (S.P.)

**Keywords:** triple negative breast cancer, organotin, mesoporous silica nanoparticles, MDA-MB-231, theranostic nanomaterials, nanobiotechnology, molecular imaging

## Abstract

Three different multifunctional nanosystems based on the tethering onto mesoporous silica nanoparticles (MSN) of different fragments such as an organotin-based cytotoxic compound Ph_3_Sn{SCH_2_CH_2_CH_2_Si(OMe)_3_} (MSN-AP-Sn), a folate fragment (MSN-AP-FA-Sn), and an enzyme-responsive peptide able to release the metallodrug only inside cancer cells (MSN-AP-FA-PEP-S-Sn), have been synthesized and fully characterized by applying physico-chemical techniques. After that, an in vitro deep determination of the therapeutic potential of the achieved multifunctional nanovectors was carried out. The results showed a high cytotoxic potential of the MSN-AP-FA-PEP-S-Sn material against triple negative breast cancer cell line (MDA-MB-231). Moreover, a dose-dependent metallodrug-related inhibitory effect on the migration mechanism of MDA-MB-231 tumor cells was shown. Subsequently, the organotin-functionalized nanosystems have been further modified with the NIR imaging agent Alexa Fluor 647 to give three different theranostic silica-based nanoplatforms, namely, MSN-AP-Sn-AX (AX-1), MSN-AP-FA-Sn-AX (AX-2), and MSN-AP-FA-PEP-S-Sn-AX (AX-3). Their in vivo potential as theranostic markers was further evaluated in a xenograft mouse model of human breast adenocarcinoma. Owing to the combination of the receptor-mediated site targeting and the specific fine-tuned release mechanism of the organotin metallodrug, the nanotheranostic drug MSN-AP-FA-PEP-S-Sn-AX (AX-3) has shown targeted diagnostic ability in combination with enhanced therapeutic activity by promoting the inhibition of tumor growth with reduced hepatic and renal toxicity upon the repeated administration of the multifunctional nanodrug.

## 1. Introduction

Breast cancer is the most common cancer in women worldwide and also the leading cause of cancer death in women (15.0%) [1,2,3]. Within all the possible different subtypes, triple negative breast cancer (TNBC) is the most aggressive subclass and it is characterized by having the worst prognosis with a high risk of relapse [2,4,5,6]. TNBCs are characterized by the absence of estrogen and progesterone receptors, as well as by having normal levels of HER2 (human epidermal growth factor receptor 2). For these reasons, hormone therapies and treatments targeting the HER2 receptor cannot be used to treat this type of cancer [7].

To tackle this issue and increase the specificity of the treatment, it is important to identify a proper receptor that meets some crucial parameters: it must be overexpressed, should not be released into circulation [8], and must become active on the target cell surface, as quickly as possible, in order to recognize more drug, to finally enhance the treatment efficiency [9]. Within the receptor that meet these criteria, the folate receptor alpha (a glycosylphosphatidylinositol (GPI) anchored cell surface glycoprotein that is able to bind free folate with high affinity) represents a good candidate being overexpressed by many cancer tissues (ovarian, epithelial, cervical, lung, kidney, brain, colorectal, and breast tumors) [10,11,12]. More specifically, the folate receptor alpha is overexpressed in many TNBCs cells, including the MDA-MB-231 cell line [13,14,15,16].

Dating from the approval of cisplatin from FDA (1978), the use of metal-based drugs in cancer chemotherapy has considerably increased [17,18,19]. Although cisplatin derivatives have been extensively used as chemotherapeutics, the onset of tumor drug-resistance has been observed upon treatment with these compounds [20]. In addition, other reported side-effects as low bioavailability and poor solubility in physiological media are still hampering the definitive application of these chemotherapeutic drugs [21]. Therefore, the search for alternative metallodrugs bearing different elements such as gallium, titanium, palladium, gold, cobalt, ruthenium, or tin could represent an interesting strategy to overcome the cisplatin-related drawbacks previously detailed [22,23]. Within the possible alternatives, organotin(IV) derivatives are getting attention as promising chemotherapeutics because of their cytotoxicity [21,24]. In addition, organotin(IV) compounds are interesting because of their potential ability to overcome resistance [25] and because they are not substrates of the P-glycoprotein 1 (permeability glycoprotein, or Pgp, also known as multidrug resistance protein 1 (MDR1)), a cell membrane transporter protein (responsible for the out of cell efflux of most of the anticancer drugs in particular) [22]. Despite its promising potential, even the organotin-based drugs present some limitations that make mandatory the use of a proper vector for their biomedical application. In this sense, nanomaterials can act as suitable vectors for metallodrug delivery by (i) protecting the active species from degradation, (ii) enhancing their therapeutic activity [26], (iii) increasing drug bioavailability and specificity, or (iv) increasing the solubility [23,27,28,29,30]. Because of its synthetic flexibility and biocompatibility, mesoporous silica-based nanostructured materials (MSN) represent one of the most used nanovectors in biomedicine, with special importance in metallodrug-based drug delivery systems [23,31,32,33,34,35,36,37,38]. Consequently, the MSNs have stood out in different biomedical fields such as molecular imaging (fluorescence and magnetic resonance imaging) and drug delivery [39,40,41,42,43,44,45]. Especially in this last research area, MSN have shown a lots of benefits [46] such as a variable and controllable particle and pore size, a large surface area that can be selectively functionalized for drug delivery or high biocompatibility [47,48,49] or the possibility of combining several functionalities in a single nanosystem [50]. In this sense, one of these possible combinations is represented by the *theranosis* or the generation of a single nanoentity able to combine therapeutic and diagnostic features at the same time [28,51,52,53,54,55]. In general, nanovectors may deliver their therapeutic cargo via two possible pathways: enhanced permeability and retention (EPR) effect [56] or receptor-mediated transcytosis [57]. In comparison with EPR, the receptor -mediated strategy is able to promote a more efficient and selective delivery of therapeutic drugs, for example, to tumoral cells.

Besides the theranostic behavior and the selective targeting ability, an ideal multifunctional nanosystem should be also able to release its therapeutic cargo in a controlled manner, for example, upon precise endogenous stimuli. Consequently, the drugs will only be accumulated in the targeted site and in a selective manner, thus, avoiding adverse side effects related to a possible off-target toxicity. For this purpose, in the last decade, stimuli-responsive “smart” nanomaterials have attracted great attention as promising materials in comparison to the conventional ones [58,59]. Within all the available “smart” materials, the enzyme-responsive ones, or the materials whose chemical structures or physical properties are responsive to the biocatalytic action of specific enzymes, have attracted great attention [59,60]. Because of dysregulation of enzyme expression in many diseases, these dysregulated enzymes can be turned into promising and selective biological triggers in therapeutics [58,61,62].

Thus, in our study, we have designed, synthesized, and fully characterized in vitro and in vivo a theranostic silica-based nanoplatform bearing therapeutic (organotin(IV) complex), diagnostic (Alexa Fluor 647) and targeting (folate fragment) moieties potentially useful in TNBCs treatment. Furthermore, we have also evaluated the effect of a site-selective controlled delivery of the metallodrug, based on the specific activation of an enzyme-responsive linker that can be cleaved only after tumor cell uptake. The synthesized materials have been fully characterized by chemical and physical techniques. The nanovectors showing the best properties have been used for in vitro toxicity evaluation. Finally, the best candidates have been assessed in vivo as theranostic agents for the metallodrug-based treatment of triple negative human breast adenocarcinoma generated in murine models. The overall achieved results were very promising paving the way for a more extensive application of these nanovectors for cancer theranosis.

## 2. Results and Discussion

### 2.1. Synthesis of the Different MSN-Based Multifunctional Nanomaterials

**Scheme 1:**
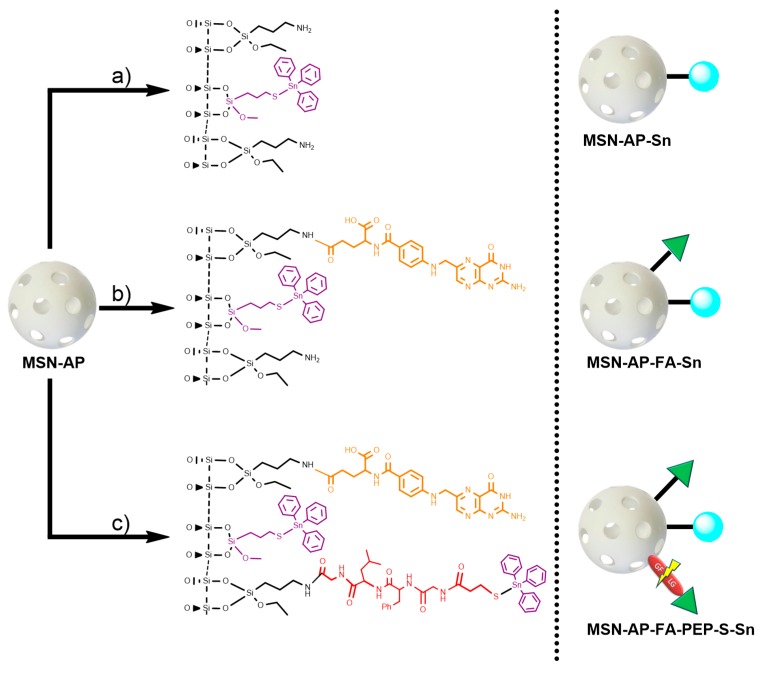
General synthetic strategy for the preparation of mesoporous silica nanoparticles (MSN) materials. Route (**a**): preparation of MSN modified with organotin metallodrug; Route (**b**): preparation of MSN modified with organotin metallodrug and folate targeting group; Route (**c**): preparation of MSN modified with organotin metallodrug, folate targeting group, and organotin metallodrug linked to enzyme-responsive peptide GFLG.

To prepare and assess the different silica-based multifunctional nanomaterials, first, mesoporous silica nanoparticles, as common nanoplatform, were synthesized as previously described and detailed here in Supporting Information. After that, the achieved MSN material was dehydrated by treatment at 150 °C under vacuum for 6 h for the subsequent surface functionalization with 3-(aminopropyl)triethoxysilane (AP) to give the MSN-AP material. For the further functionalization steps, the MSN-AP material was then functionalized with an organotin-based metallodrug by using three different strategies, one for the creation of each required moiety (Scheme 1).

In more details, the first designed method consisted in the direct tethering reaction of MSN-AP with Ph_3_Sn{SCH_2_CH_2_CH_2_Si(OMe)_3_} complex via protonolysis and elimination of methanol groups to obtain the material MSN-AP-Sn (Scheme 1, route a). The resulting material is likely to be cytotoxic, as our group has demonstrated that Sn-functionalized silica-based nanostructured show interesting cytotoxic properties in other cancer cell lines [34,36]. The second strategy expected the incorporation of the organotin metallodrug after the previous functionalization of MSN-AP material with a folate fragment, in order to study the impact of the potential receptor-mediated active targeting ability on the biological properties of the nanomaterial (Scheme 1, route b). The incorporation reaction of targeting moiety was carried out by coupling the folic acid with the amino groups of the MSN-AP owing to the carbodiimide chemistry and using EDAC as coupling agent. The material with folic acid (MSN-AP-FA) was subsequently reacted with Ph_3_Sn{SCH_2_CH_2_CH_2_Si(OMe)_3_} in a tethering reaction to give the material MSN-AP-FA-Sn via the elimination of methanol groups of the tin compound. Again, this material is expected to show cytotoxic activity because of the incorporation of the highly cytotoxic SnPh_3_ moiety and the potential higher uptake due to the incorporation of the folic acid fragment.

Finally, the third strategy was designed in order to anchor the metallodrug to the nanovector surface by means of an enzyme responsive linker (ERL) based on a specific peptide sequence. The selected GFLG tetrapeptide linker (highly sensitive to the lysosomal cysteine protease cathepsin B that is overexpressed in breast adenocarcinoma) [63] will presumably permit the release of the therapeutic cargo (organotin(IV) metallodrug), once the theranostic nanovector is uptaken by the cancer cells [64,65]. Toward this scope, the MSN-AP material was treated simultaneously with folic acid and the Fmoc-GFLG-COOH protected peptide by an EDAC-assisted coupling reaction in order to achieve the material MSN-AP-FA-PEP. Subsequently, the terminal amino group of the peptide was deprotected with pyrrolidine and reacted with 3-mercaptopropionic acid to give the thiol-pendant system MSN-AP-FA-PEP-S. Thus, the latter was finally reacted with Ph_3_Sn{SCH_2_CH_2_CH_2_Si(OMe)_3_} complex in the presence of an excess of triethylamine to give the final material MSN-AP-FA-PEP-S-Sn (Scheme 1, route c). In this material, the triphenyltin fragments are present both directly attached to the silica-material (as in the case of MSN-AP-Sn and MSN-AP-FA-Sn systems) and also attached to the modified peptide via a Sn-S bond formed with the thiol group of the added mercaptopropionic acid.

### 2.2. Physical and Chemical Characterization of the Different MSN-Based Multifunctional Nanomaterials

For the characterization of the silica-based nanomaterials, different solid-state techniques were used. First, FT-IR studies were carried out to identify each fragment attached to the silica system during the modifications. Thus, for example, the IR spectrum of the nanomaterial functionalized with the organotin complex and folic acid (MSN-AP-FA-Sn, Figure S1 of Supporting Information) shows the typical bands of a functionalized silica-based nanoparticle, namely, a broadband between 3500–3200 cm^−1^ attributed to the O-H bonds of free silanol groups and the absorbed water in the silica. In addition, a strong band at 1100 cm^−1^ corresponding to the silanol groups (Si-O-Si) was observed. Furthermore, a medium intensity band at 900 cm^−1^ was presented and assigned to the stretching bands of Si-O bonds. Interestingly, a set of different low intensity bands were observed between ca. 3100 and 2800 cm^−1^ and attributed to the C-H and N-H vibrations of the different ligands (AP and FA). Finally, between 1700 and 1300 cm^−1^ and 680–740 cm^−1^ low intensity different bands associated with all the amido and carbonyl groups were also observed confirming the presence of the different functionalizing fragments. A similar spectrum was also observed for the material MSN-AP-FA-PEP-S-Sn (Figure S2 of Supporting Information).

**Figure 1:**
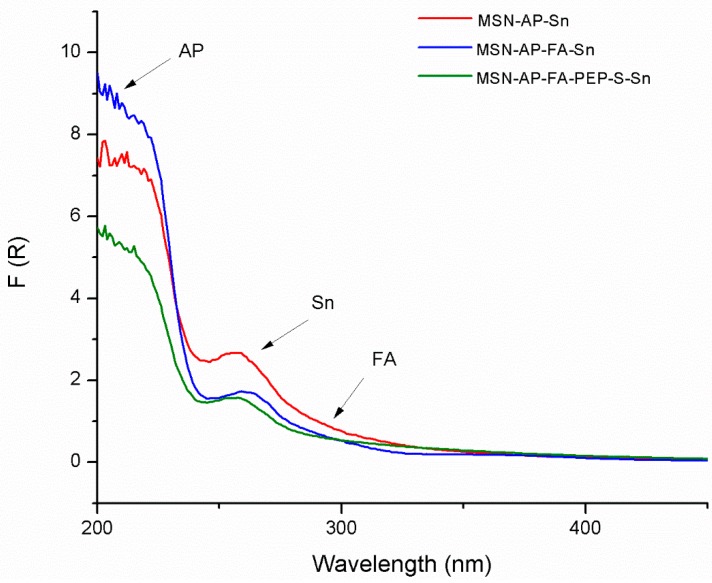
Diffuse reflectance ultraviolet spectroscopy (DR-UV) spectra of the tin-functionalized materials MSN-AP-Sn, MSN-AP-FA-Sn, and MSN-AP-FA-PEP-S-Sn.

The functionalized materials were also characterized by diffuse reflectance ultraviolet spectroscopy (DR-UV), an especially useful technique for the characterization of the absorption of the different functionalizing ligands or metallodrugs in silica-based systems in solid state (Figure 1). In all cases, the tin-containing materials showed a very intense peak between 200 and 220 nm due to the amino ligand AP and at ca. 260 nm due to the functionalization with the organotin derivate. Interestingly, the DR-UV spectra of both MSN-AP-FA-Sn and MSN-AP-FA-PEP-S-Sn showed an additional low intensity shoulder at ca. 320 nm, which is attributed to the anchored folic acid of the material (Figure 1).

For the quantification of the amount of functionalization, TG studies were carried out for all the functionalized materials, showing a similar mass loss close to 14% between 110 °C and 700 °C, which indicates a similar functionalization degree (Table S1 and Figure S3 of Supporting Information). In addition, XRF studies of the tin-containing materials showed that, relying on the observed S:Sn ratio of approximately 1:5, it seems that not all the tin is linked to MSN through the mercaptopropyl ligand in MSN-AP-Sn and MSN-AP-FA-Sn, but the migration of SnPh_3_^+^ fragments and formation of species of the type Si-O-SnPh_3_ also occurred. Interestingly, in the case of MSN-AP-AF-PEP-S-Sn material, the S:Sn ratio lowered up to 1:2.7 indicating that the formation of the Si-O-SnPh_3_ species is minimized and the SnPh_3_ complex is reacting with both the mercaptopropyl group and the thiol group of the modified peptide (Table S2 of Supporting Information).

**Figure 2:**
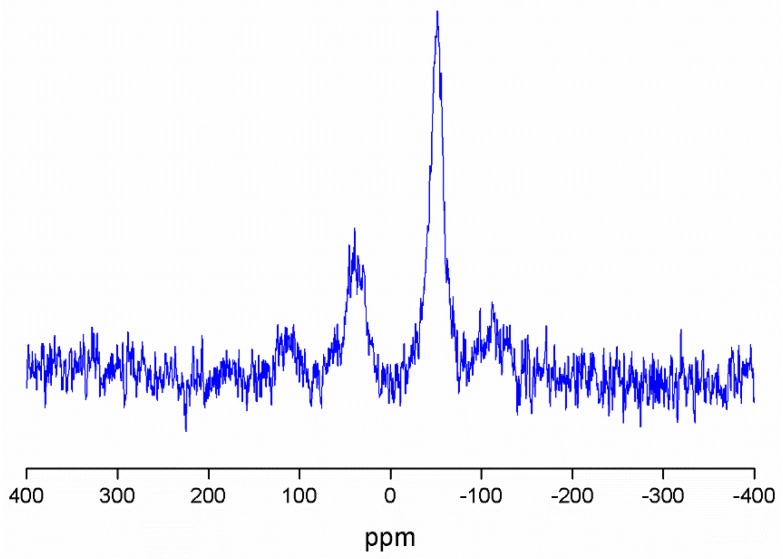
^119^Sn MAS NMR spectrum of MSN-AP-FA-Sn.

The MSN-AP-AF-Sn (Figure 2) and MSN-AP-AF-PEP-S-Sn (Figure S4) were also characterized by ^119^Sn MAS NMR spectroscopy. In both cases, the spectra are characterized by the appearance of two broad signals at ca. −52 ppm and 40 ppm. The signal recorded at −52 ppm corresponds to the tin atom bound to the S of either the mercaptopropyl ligand or of the thiol-modified peptide, while the signal at ca. 40 ppm is due to the tin atoms of the Si-O-SnPh_3_ species.

**Figure 3:**
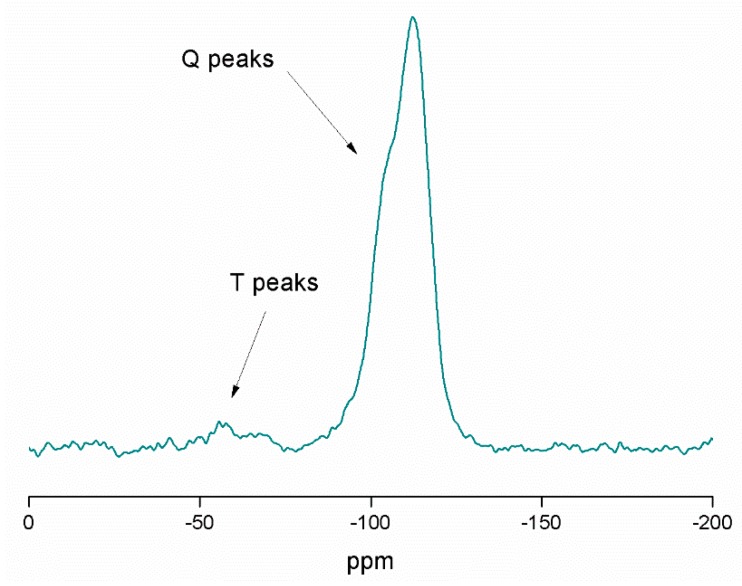
^29^Si MAS NMR spectrum of MSN-AP-FA-PEP-S-Sn.

In addition, ^29^Si MAS NMR spectroscopy was used for the characterization of the tin-containing material MSN-AP-FA-PEP-S-Sn (Figure 3). The spectrum mainly shows the signals of the silicon atoms of the structure, such as [Si(OSi)_2_] (the highest peak (Q^4^)) at −112.7 ppm), [Si(OSi)_2_(OH)] (a very intense peak Q^3^ at −105.3 ppm), [Si(OSi)_2_(OH)_2_] (a low intensity peak (Q^2^) at −95.3 ppm), and the [Si(OSi)(OH)_3_] (Q^1^, very difficult to observe at −86.2 ppm because of its low intensity). The high intensity of Q^4^ and Q^3^ is in agreement with the expected for the mesoporous nature of the synthesized silica nanoparticles. In addition, at ca. −69 and −62 ppm, the T^2^ ((SiO)_2_SiOH–R) and T^3^ ((SiO)_3_Si–R) peaks of very low intensity were also observed. In addition, the different spectra of the intermediate materials MSN-AP (Figure S5 of Supporting Information) and MSN-AP-FA-PEP (Figure S6 of Supporting Information), show slight changes on the intensity of the different Q^4^, Q^3^, and Q^2^ peaks, as well as the T^2^ and T^3^ peaks. This indicates that the different functionalization reactions do not have a strong influence on the chemical environment associated with the silicon atoms of the material.

Beside a deep physico-chemical characterization, the tin-containing synthesized materials were also characterized in terms of size and morphological appearance by transmission electron microscopy (TEM), as well as by dynamic light scattering (DLS).

**Figure 4:**
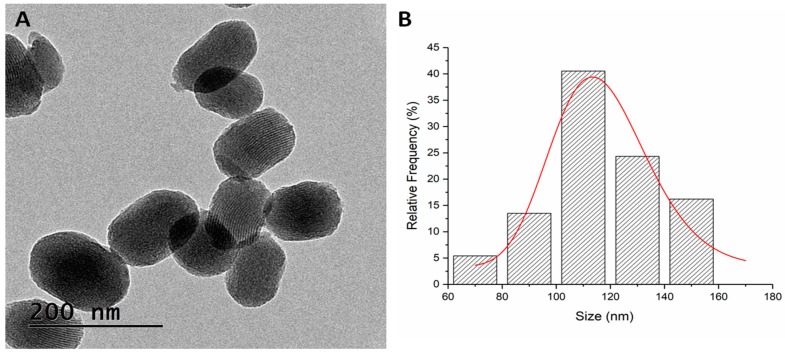
TEM micrograph of MSN-AP-AF-Sn (**A**) and mean size distribution (**B**).

The TEM micrograph retrieved oval-shaped nanoparticles with a mean particle size distribution of 116.15 ± 3.67 nm (Figure 4).

**Figure 5:**
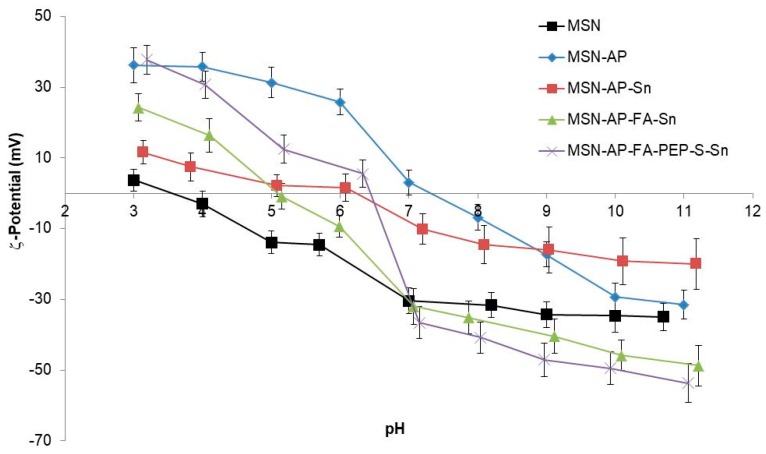
Z-Potential of the different functionalized nanoparticles.

The DLS-mediated hydrodynamic size measurements of the MSN multifunctional nanomaterials were 290 (PDI: 0.287), 312 (PDI: 0.274) and 323 (PDI: 0.282) nm for MSN-AP-Sn, MSN-AP-FA-Sn, and MSN-AP-FA-PEP-S-Sn, respectively. The surface ζ-potential of the MSN nanomaterials resulting from each synthetic step was in agreement with the expected values (Figure 5). As general consideration, all the synthesized nanomaterials showed an isoelectric point (pI) value lower than the physiological pH (≈7.4) (Table S3, Supporting Information). For example, the pI values for the final MSN-AP-FA-Sn and MSN-AP-FA-PEP-S-Sn materials were 5.1 and 6.4 respectively. This behavior will grant a surface negative charge of metallodrug-functionalized silica-based nanomaterials once administered in physiological environment. The latter is a crucial requirement in order to confer a good colloidal stability to the nanoparticles.

### 2.3. In Vitro Characterization of the Different MSN-Based Multifunctional Nanomaterials

**Figure 6:**
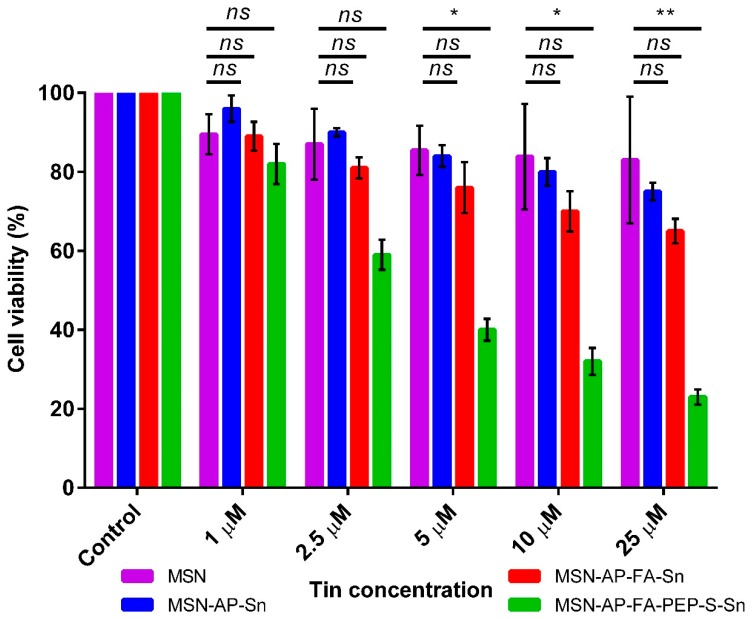
MTT viability assay of MDA-MB-231 cells after incubation with different concentrations of the MSN nanomaterials. All the results were expressed as % of control (mean ± SD, n = 3 independent experiments and 3 replicates/experiment). Significance was calculated by one-way ANOVA Tukey’s test. ns: *p* > 0.05 or not significant statistical difference between the groups of data; *: *p* < 0.05 or significant statistical difference between the groups of data; **: *p* < 0.01 or significant statistical difference between the groups of data.

After the successful deep characterization of all the achieved multifunctional MSN materials, their newly generated therapeutic properties were characterized in a different set of in vitro analyses. First, the MTT cell viability assay was carried out in order to assess the ability of all MSN materials to inhibit the viability of MDA-MB-231 human breast cancer cells as a function of the concentration (Figure 6). The results clearly indicate that, at all the tested concentrations, the MSN-AP-FA-PEP-S-Sn material functionalized with the ERL-peptide was able to reduce the cell viability in a more efficient manner in comparison with the nanoparticles showing the metallodrug directly linked to the silica material (Figure 6). Furthermore, it can also be observed that the viability decreased in an inverse proportional manner with respect of the tin concentration rise. Thus, these results clearly confirmed the correct design of the peptide-based controlled delivery system created for this multifunctional silica-based nanodrug. In fact, the anti-proliferative activity enhancement of organotin metallodrug MSNs was achieved only in the case of interaction with tumor cells from breast adenocarcinoma that have been described overexpressing both folate receptor alpha and the cathepsin B enzyme, being this last the responsible of peptide cleavage and the metallodrug release [59,66,67].

**Figure 7:**
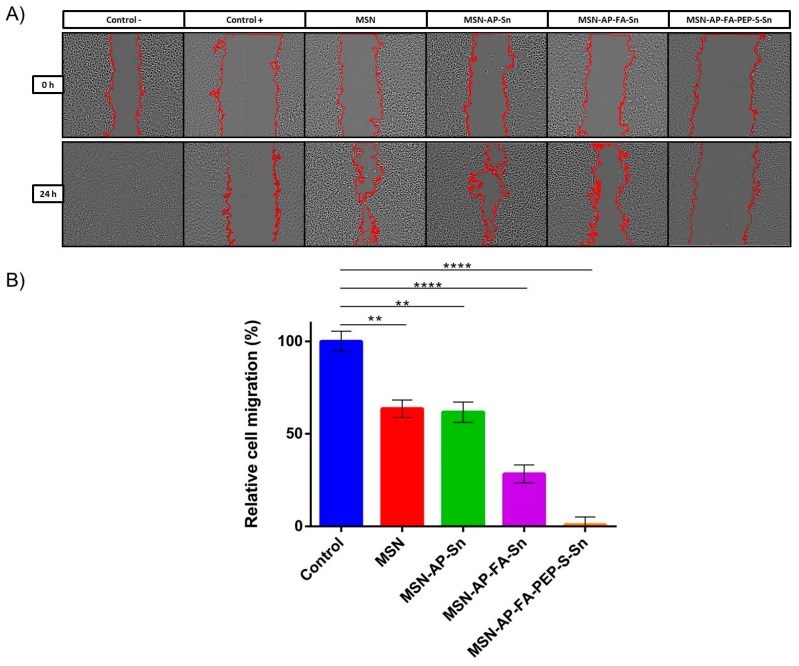
Wound healing assay of MDA-MB-231 cells incubated with the MSN nanoparticles. (**A**) Phase contrast microscopy images of wells. The edges of the scar are marked in red. Positive control (control +): cells incubated with DMSO (complete migration inhibition); negative control (control −): cells grown in culture media (complete migration and wound healing). (**B**) Quantification of cell migration. The results were expressed as % of the initial scar area (mean ± SD, n = 2 independent experiments and 2 replicates/experiment)**.** Significance was calculated by unpaired t-test of one-way ANOVA. **: *p* < 0.01 or significant statistical difference between the two groups of data; ****: *p* < 0.0001 or highly significant statistical difference between the two groups of data.

Besides the antiproliferative activity, we decided to evaluate also the potential inhibition of migration ability of MDA-MB-231 cells by means of wound healing assay (Figure 7). This is a standard in vitro technique for probing collective cell migration in two dimensions that is also known as sheet migration. This migration behavior typically occurs in diverse processes such as cancer metastasis [68,69,70], embryonic morphogenesis [71], and tissue injury [72]. In order to ensure the characterization of the potential anti-migration ability without promoting cell death, based on the results achieved previously, we decided to carry out this assay using 1 μM tin concentration per well or the lowest toxic concentration identified by MTT assay (Figure 6). The results obtained with this assay indicate a general positive trend about the inhibition of cell migration related to the use of each material (Figure 7). The obtained relative inhibition percentage has been calculated by comparing the residual scar areas after 24 h edge progression vs. the initial gap area. As reported in Figure 7, the cells incubated with bare MSN and only tin-functionalized nanomaterials are able to promote the wound healing in major percentage (63.55% and 62% for MSN and MSN-AP-Sn, respectively) in comparison with the control cells. In other terms, in absence of folate targeting, the MDA-MB-231 cell migration is only moderately inhibited. Conversely, the cells treated with both folate-functionalized materials are much more inhibited during their gap closure. In fact, especially in the case of MSN-AP-FA-PEP-S-Sn material, the wound healing after 24 h was almost completely negligible indicating a clear inhibitory effect on the migration mechanism of MDA-MB-231 tumor cells that is directly related to the overall design of our nanodrug (combination of active targeting *plus* ERL-mediated controlled release mechanisms) (Figure 7).

**Scheme 2:**
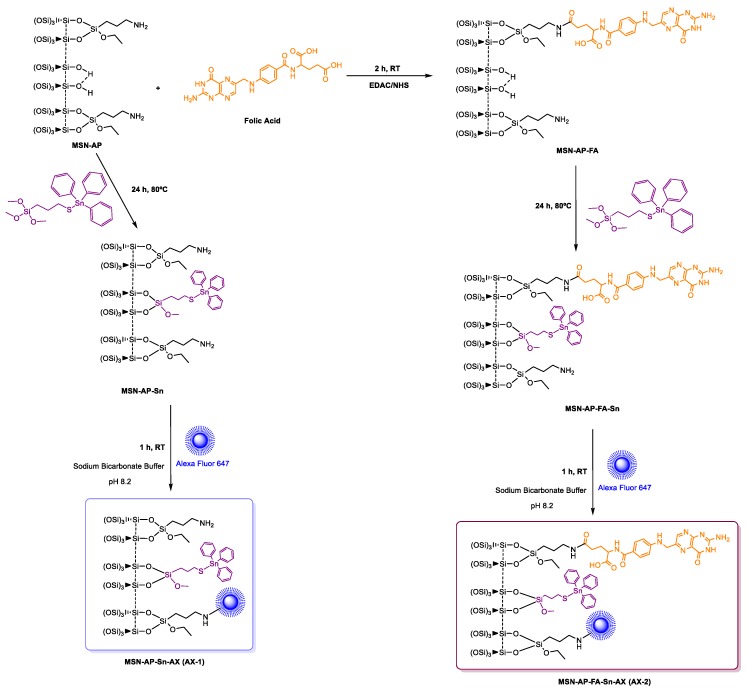
Reactions of the formation of the theranostic tin-functionalized materials AX-1 and AX-2.

**Scheme 3:**
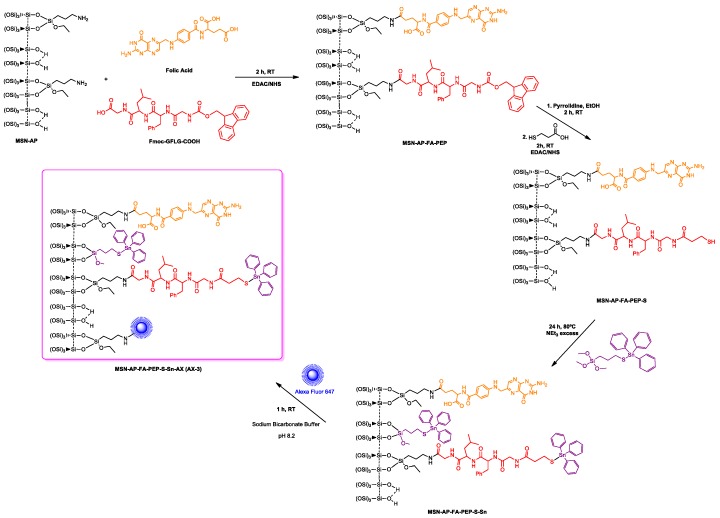
Reactions of the formation of the tin-functionalized material AX-3.

After the positive evaluation of therapeutic potential of our metallodrug-based MSN nanoplatform, we decided to go further and evaluate its imaging ability, a crucial requirement for the potential in vivo application. Toward this scope, we functionalized the nanomaterials with Alexa Fluor 647 dye (AX), in order to enable NIR fluorescence imaging in vivo. The chemical anchoring of the dye was carried out by carbodiimide chemistry as per the manufacturer’s instructions, in a reaction of the different tin-functionalized materials MSN-AP-Sn, MSN-AP-FA-Sn, or MSN-AP-FA-PEP-S-Sn to give AX-1, AX-2, and AX-3 materials, respectively (Scheme 2 and Scheme 3).

The outcome of the dye functionalization reaction was successfully verified by FTIR (Figure S2 of Supporting Information) and DR-UV spectroscopy (Figure S7 of Supporting Information) of labeled nanoparticles. In addition, fluorescence imaging of MSN nanomaterials after coupling reaction with NIR dye Alexa Fluor 647 was carried out to confirm the correct incorporation of the fluorophore (Figure S8 of Supporting Information).

**Figure 8:**
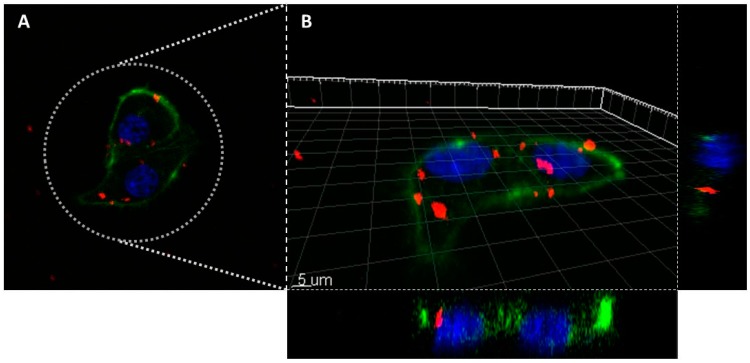
Confocal laser scanning microscope images of the MDA-MB-231 cells incubated overnight with the nanomaterial AX-3 (**A**): 2D image; (**B**): 3D reconstruction). Colors legend: MSN nanoparticles are in red (Alexa Fluor 647, Ex.650/Em.665 nm), cellular nucleus are in blue (DAPI staining, Ex.358/Em.461) and actin filaments are in green (Phalloidin staining, Ex.495/Em.519 nm).

Thus, to assess effectively the in vitro imaging ability in biological environment, the MDA-MB-231 cells were incubated 24 h with AX-labeled nanoparticles of MSN-AP-FA-PEP-S-Sn-AX nanoparticles (AX-3) and the cell uptake was analyzed by confocal laser scanning microscopy (CLSM) (Figure 8). The 2D and 3D images show a clear internalization of AX-3 nanoparticles inside the tumor cells (Figure 8A,B). In addition, based on the 3D image analysis (Figure 8B), the presence of inner circular accumulations of nanoparticles can be observed. Considering the size, shape, and localization, these repetitive structures should correspond to the lysosome vesicles that are promoting the MSN cell internalization and metallodrug activation mediated by peptide cleavage at the same time [64,65].

### 2.4. In Vivo Evaluation of Theranostic Properties of Metallodrug Based MSN Nanomaterials on Breast Adenocarcinoma Mice Models

**Figure 9:**
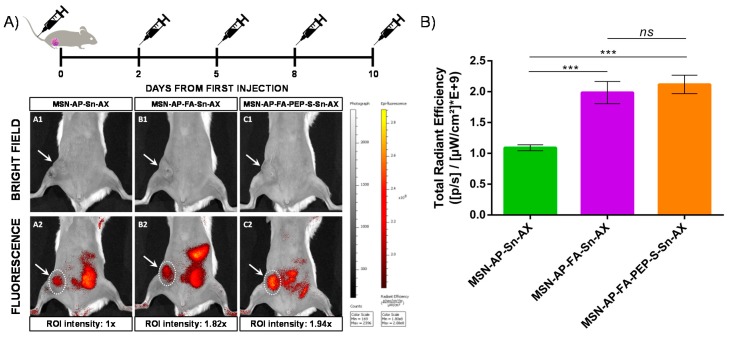
(**A**) In vivo fluorescence imaging of mice treated with different nanoparticles at endpoint: A: MSN-AP-Sn-AX (AX-1); B: MSN-AP-FA-Sn-AX (AX-2); C: MSN-AP-FA-PEP-S-Sn-AX (AX-3). Row 1 (A1, B1, and C1) corresponds to representative bright field images to visualize the tumor area (white arrows) and row 2 (A2, B2, and C2) corresponds to representative fluorescence images acquired 2 h post-injection of nanoparticles. The applied ROI areas used for signal intensities comparison are all equal and marked with white dotted lines. (**B**) Total in vivo fluorescence accumulated in the mice tumor treated with different nanomaterials at end point (*n* = 3 for each group). The applied ROI area was the same in each case. Significance was calculated by unpaired t-test of one-way ANOVA. ns: *p* > 0.05 or not significant statistical difference between the groups of data; ***: *p* < 0.001 or highly significant statistical difference between the two groups of data.

After the in vitro evaluation of the therapeutic and imaging properties of MSN nanoplatforms, the assessment of their potential theranostic activity in vivo was carried out. Toward this scope, human breast adenocarcinoma bearing mice were used. The mice were randomized in four different groups (*n* = 3 each group) in order to assess (i) the active targeting contribution vs. passive tumor adsorption mediated by the EPR effect, (ii) the therapeutic activity of Sn directly linked on MSN surface vs. (iii) the controlled delivery of organotin metallodrug promoted by lysosomal cleavage of ERL-peptide and (iv) a control group treated with saline. The different MSN nanomaterials were administered 5 times during 10 days by tail vein injection by following the posology timeline reported in Figure 9.

First, the diagnostic properties were evaluated by tracking the nanoparticle biodistribution 2 h post-injection of each dose by in vivo fluorescence imaging (IVIS) of mice full body (Figure 9A). The representative images of mice treated with the three different MSN theranostic nanoparticles (MSN-AP-Sn-AX (AX-1), MSN-AP-FA-Sn-AX (AX-2), and MSN-AP-FA-PEP-S-Sn-AX (AX-3)) and acquired at the end of the whole treatment showed clearly an accumulation within the tumor mass (white arrows) as well as in the liver and in the bowel, confirming the preferential excretion route. After analyzing and normalizing the intensities of the same region-of-interest (ROI) areas centered on the tumor zones for each treated mouse (white spotted line), the accumulation efficiency is almost two folds higher for both folate-targeted nanoparticles (AX-2 and AX-3) in comparison with the passively diffused ones (AX-1)(Figure 9B). These results confirmed the successful folate receptor-targeted activity of the organotin-based MSN nanovectors.

**Figure 10:**
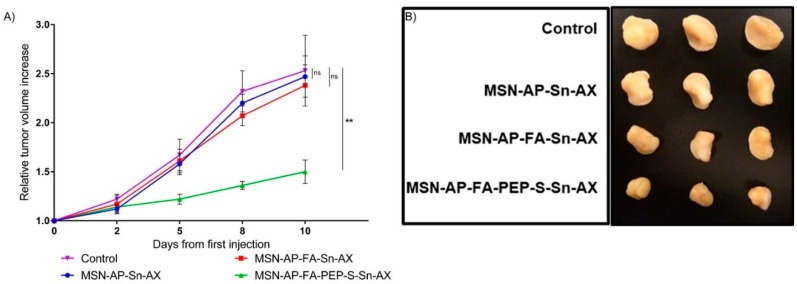
(**A**) Relative tumor volume increase over the course of the treatment with the nanomaterials or saline (control) (n = 3 mice/group). Significance was calculated by unpaired t-test of one-way ANOVA. ns: *p* > 0.05 or not significant statistical difference between the groups of data; **: *p* < 0.01 or significant statistical difference between the two groups of data; (**B**) ex vivo tumor images for all mice treated with different MSN nanomaterials or saline (control).

After assessing successfully the targeted diagnostic ability of MSN nanomaterials, we then evaluated also their therapeutic potential. In this sense, the tumor mass growth of the mice groups treated with all the MSN nanomaterials was analyzed by checking their dimensions during the entire treatment. The achieved results, reported as the relative volume increase of all the tumor mass at the end of each treatment and normalized according to the initial tumor mass at the beginning of each treatment, are summarized in Figure 10A.

After 10 days of nanoparticle administration, the tumor mice groups treated with saline or the MSN-AP-FA-Sn-AX underwent an average tumor mass increase by 2.5 and 2.4 folds respectively in comparison with the volume of each tumor mass measured at the beginning of the nanotherapy. Conversely, the breast tumors of the mice treated with MSN-AP-FA-PEP-S-Sn-AX nanoparticles only increased their volumes by 1.5 folds on average (Figure 10A). The Figure 10B shows the ex vivo images of the tumor proceeding by each experimental group (saline, MSN-AP-Sn-AX, MSN-AP-FA-Sn-AX, and MSN-AP-FA-PEP-S-Sn-AX nanoparticle, respectively) after postmortem excision. Photographs and weight of excised tumor at endpoint confirmed that only MSN-AP-FA-PEP-S-Sn-AX (AX-3) treatment inhibited tumor growth (Figure 10B and Figure S9).

Altogether, all these results successfully demonstrate the therapeutic potential against breast adenocarcinoma of the organotin-based theranostic nanoplatform as well as the effectiveness and relevance of the overall nanoplatform design. In fact, the concomitant use of folate as targeting agent together with ERL-peptide for controlled drug delivery has been demonstrated to be crucial in order to allow the cytotoxic organotin fragment to be cleaved from the nanostructure selectively inside the tumoral cell and then to exert its chemotherapeutic effect.

Finally, the long-term in vivo toxicity of the nanomaterials was checked by monitoring the possible effects on the liver and kidney functions both being the principal excretion routes. To this scope, the serum level of alanine aminotransferase (ALT), aspartate aminotransferase (AST), alkaline phosphatase (AKP), albumin (ALB), and blood urea nitrogen (BUN) were monitored before and after each treatment (Figure S10). The quantification of these enzymes in serum is generally considered a useful biomarker to control the hepatic and renal functionalities. In fact, increased levels of these biomarkers are generally associated to acute and/or chronic liver and kidney injuries. As reported in Figure S10, the treated vs. control serum levels of all hepatic and renal biomarkers were kept constant or slightly decreased for all the mice groups at the end of each nanotherapy. Consequently, these results indicate that no liver or kidney damage has been caused in vivo because of the administration of the organotin metallodrug over the course of the treatment. Nevertheless, we are aware about the necessity to deeply analyze in forthcoming works especially the potential liver injury risk in humans. Nonetheless, based on the achieved overall results, our hypothesis of the potentially limited in vivo toxicity thanks to the combination of the targeting ability of the nanosystem *plus* the selective enzymatic metallodrug activation, has been successfully confirmed in our mouse model.

## 3. Materials and Methods

### 3.1. Synthesis of Nanomaterials

#### 3.1.1. General Conditions

All reactions were performed using standard Schlenk tube techniques in an inert atmosphere of dry nitrogen. Solvents were distilled from the appropriate drying agents and degassed before use. The reagents used in the preparation of the starting material (MSN), tetraethyl orthosilicate (TEOS) and hexadecyltrimethylammonium bromide (CTAB) were purchased from Sigma Aldrich and Acros Organics, respectively. The ligand 3-(aminopropyl)triethoxysilane (AP) was purchased from Sigma Aldrich, and 3-mercaptopropyltrimethoxysilane (MP) from Fluorochem. 3-Mercaptopropionic acid, triphenyltin chloride, folic acid, and all the reagents necessary for EDAC coupling, were purchased from Sigma Aldrich. AlexaFluor^TM^ 647 NHS ester, tris(trithlamonium salt) from Invitrogen. The Fmoc-GFLG-COOH peptide was custom made synthesized by Biomedal (Sevilla, Spain).

#### 3.1.2. Synthesis of the Starting Material: Mesoporous Silica Nanoparticles (MSN)

The synthesis of MSN was carried out from a modification of the experimental procedure reported by Zhao et al. [73], using CTAB (1 g, 2.74 mmol) as surfactant in a basic solution of nanopure water and TEOS (5 mL, 22.4 mmol) as silicon source. The reaction was carried out during 2 h at 80 °C. After that time, the precipitate was dried and calcined to 550 °C for 16 h to obtain the starting mesoporous material MSN.

#### 3.1.3. Functionalization with Amino Ligands. Preparation of MSN-AP Material

The functionalization with amino ligands was carried out with a slight modification of reported procedures [74]. In brief, the starting material (MSN) was dehydrated at 80 °C under vacuum for 24 h. Subsequently, MSN was suspended in dry toluene and a defined amount of the amino ligand (AP) was added to the mixture. The suspension was then stirred at 110 °C for 48 h. Afterwards the mixture was then centrifuged, washed with toluene (2 × 10 mL) and diethylether (2 × 10 mL).

#### 3.1.4. Functionalization with the Cytotoxic Agent (Sn). Synthesis of MSN-AP-Sn

**Scheme 4:**
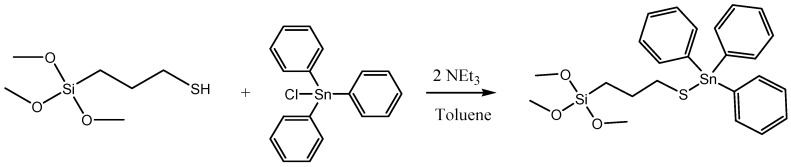
Reaction of the formation of the cytotoxic organotin(IV) derivative.

First, the formation of the organotin derivate Ph_3_Sn{SCH_2_CH_2_CH_2_Si(OMe)_3_} was carried out via the reaction of triphenyltin(IV) chloride and 3-mercaptopropyltrimethoxysilane (Scheme 4). In a Schlenk tube, SnPh_3_Cl (284.1 mg) was dissolved in dry toluene in a proportion of 20% wt Sn/SiO_2_. Subsequently, 3-mercaptopropyltrimethoxysilane (178.1 μL) and triethylamine (205.5 μL) was added in a relation of 1:1:2 molar respectively regarding the organotin compound. The solution was kept under stirring and nitrogen atmosphere during 24 h at 80 °C. After that time, the stirring was stopped, and the compound was filtered and added, under nitrogen and with a cannula, to a toluene suspension of the silica material MSN-AP. The reaction mixture was stirred at 80 °C for 24 h. The material was then filtered and washed with toluene (2 × 10 mL) and diethylether (2 × 10 mL).

#### 3.1.5. Incorporation of Folic Acid (FA). Synthesis of MSN-AP-FA

For the incorporation of folic acid in the amino-functionalized material MSN-AP an EDAC coupling reaction was carried out (Scheme 2). In a general method, 18.4 mg of folic acid (5% weight with respect to the quantity of MSN-AP) was dissolved in dimethylsulfoxide (DMSO) and subsequently added to 35 mL of a 0.1 M of MES buffer. EDAC (14 mg) and NHS (21 mg) were added to the mixture and reacted under vigorous stirring for 15 min at room temperature. Subsequently, 350 mg of MSN-AP was added to the EDAC solution and reacted at room temperature under stirring. Then, 2-mercaptoethanol (55.5 μL) was added to the mixture and the suspension was stirred for 2 additional hours. Finally, the reaction mixture was centrifuged, washed with DMSO, ethanol, water, and dried overnight in a stove at 80 °C. Finally, 300 mg of the material MSN-AP-FA was obtained.

#### 3.1.6. Functionalization with the Cytotoxic Agent (Sn). Synthesis of MSN-AP-FA-Sn

The functionalization with the cytotoxic tin agent Ph_3_Sn{SCH_2_CH_2_CH_2_Si(OMe)_3_} as described previously for material MSN-AP-Sn (Scheme 2), but using the following quantities: SnPh_3_Cl (243.54 mg), 3-mercaptopropyltriethoxysilane (152.62 μL) and triethylamine (176.12 μL)) and MSN-AP-FA (300 mg) (Scheme 3). Finally, 280 mg of the material MSN-AP-FA-Sn was obtained.

#### 3.1.7. Simultaneous Incorporation of Folic Acid (FA) and Targeting Peptide (Fmoc-Gly-Phe-Leu-Gly-COOH). Synthesis of MSN-AP-FA-Pep

For the simultaneous functionalization of the amino-functionalized material MSN-AP with folic acid and the peptide Fmoc-Gly-Phe-Leu-Gly-COOH (Fmoc-GFLG-COOH), an EDAC coupling reaction was performed (Scheme 3). In a general method, a quantity of folic acid (11 mg, 5% weight with respect to the quantity of MSN-AP) and Fmoc-GFLG-COOH (11 mg, 5% weight with respect to the quantity of MSN-AP) were dissolved in DMSO using an ultrasound bath and the solution was added to 42 mL 0.1 M of MES buffer. EDAC (16.8 mg) and NHS (25.2 mg) were added to the mixture and reacted under vigorous stirring for 15 min at room temperature. Subsequently, MSN-AP (210 mg) was added to the EDAC solution and reacted at room temperature under stirring. Then, 2-mercaptoethanol (74.3 µL) was added to the mixture and the suspension stirred for 2 additional hours. Finally, the reaction mixture was centrifuged, washed with DMSO (2 × 10 mL), ethanol (2 × 10 mL), water (2 × 10 mL) and dried in a stove at 80 °C overnight. Finally, 210 mg of the material MSN-AP-FA-PEP was obtained.

#### 3.1.8. Incorporation of 3-Mercaptopropionic Acid (MS). Synthesis of MSN-AP-FA-PEP-S

Before the coupling of MS, the Fmoc deprotection is needed. For the deprotection reaction a 1:3 molar suspension peptide:pyrrolidine (210 mg MSN-AP-FA-PEP and 9.3 µL of pyrrolidine) in 20 mL of ethanol was reacted during 2 h at room temperature. The solid was centrifuged, washed with ethanol, and kept in cold. For the incorporation of MS, an EDAC coupling was carried out using a very similar method than that of the synthesis of MSN-AP-FA. The synthesis consisted of the addition of one equivalent of MS for each equivalent of the anchored peptide (210 mg of deprotected MSN-AP-FA-PEP and 3.2 µL of 3-mercaptopropionic acid (MS)), in the presence of 21 mL 0.1 M of MES buffer. EDAC (8.4 mg) and NHS (12.6 mg) were also used (Scheme 3). The obtained solid was washed with DMSO (2 × 10 mL), ethanol (2 × 10 mL) and water (2 × 10 mL). The solid was then dried in a stove at 80 °C. Finally, 200 mg of the material MSN-AP-FA-MS was obtained.

#### 3.1.9. Functionalization with the Cytotoxic Agent (Sn). Synthesis of MSN-AP-FA-PEP-S-Sn

The functionalization with the cytotoxic tin agent was carried out by the addition of the SnPh_3_ moiety from the compound Ph_3_Sn{SCH_2_CH_2_CH_2_Si(OMe)_3_} which was prepared as described previously for material MSN-AP-Sn, but using the following quantities: SnPh_3_Cl (97.4 mg), 3-mercaptopropyltrimethoxysilane (61.1 μL) and triethylamine (176.1 μL). The compound Ph_3_Sn{SCH_2_CH_2_CH_2_Si(OMe)_3_} prepared in situ was added to a suspension of MSN-AP-FA-PEP-S (120 mg) in toluene in the presence of excess of triethylamine. The reaction mixture was stirred for 24 h at 80 °C (Scheme 4). The excess of triethylamine allows the deprotonation of the thiol group of the peptide and the formation of the S-SnPh_3_ moiety by an exchange reaction with the compound Ph_3_Sn{SCH_2_CH_2_CH_2_Si(OMe)_3_} leading to the formation of the material MSN-AP-FA-PEP-S-Sn. The material was filtered and washed with toluene (2 × 10 mL), ethanol (2 × 10 mL), and water (2 × 10 mL). Finally, 110 mg of the material MSN-AP-FA-PEP-S-Sn was obtained.

#### 3.1.10. Incorporation of the Imaging Agent Alexa Fluor 647. Preparation of AX Materials

For the incorporation of the fluorophore, 100 µg of Alexa-Fluor 647 were dissolved in 100 µL of dimethylsulfoxide (DMSO) and the solution was added to 50 mg of the materials functionalized with tin MSN-AP-Sn, MSN-AP-FA-Sn, or MSN-AP-FA-PEP-S-Sn dispersed in 5 mL of 0.1 M sodium bicarbonate buffer at pH 8.2. The mixture was reacted for 1 h at room temperature under dark conditions. Finally, the solid was centrifuged and washed with DMSO (2 × 5 mL) and water (2 × 5 mL) to give MSN-AP-Sn-AX (AX-1), MSN-AP-FA-Sn-AX (AX-2) or MSN-AP-FA-PEP-S-Sn-AX (AX-3), respectively.

### 3.2. General Remarks on Chemico-Physical Characterization of the Materials

X-ray diffraction (XRD) pattern of the systems were obtained on a Philips Diffractometer model PW3040/00 X’Pert MPD/MRD at 45 kV and 40 mA, using a wavelength Cu Kα (λ = 1.5418 Å). Sn wt% determination by X-ray fluorescence were carried out with an X-ray fluorescence spectrophotometer Philips MagiX with an X-ray source of 1 kW and a Rh anode using a helium atmosphere. Thermogravimetry analyses (TG) were obtained on a Shimadzu mod. DSC-50Q (Shimadzu, Kioto, Japan) operating up to 700 °C (ramp 20 °C/min) at an intensity of 50 A. IR spectra were prepared using KBr pellets with a spectrophotometer Termo Nicolet Avatar 380 FT-IR with a Michelson filter interferometer. DR UV-Vis measurements were carried out on a Varian Cary-500 spectrophotometer equipped with an integrating sphere and polytetrafluoroethylene (PTFE) as reference. ^119^Sn MAS NMR and ^29^Si MAS NMR spectra, were recorded on a Varian-Infinity Plus Spectrometer at 400 MHz for proton frequency (4 μs 90° pulse, 4000 transients, spinning speed of 6 MHz, contact time 3 ms, pulse delay 1.5 s). Transmission electron microscopy (TEM) was carried out on a JEOL JEM 1400, operating at 100 kV. Dynamic light scattering (DLS) measurements were carried out on a Zetasizer Nano Zen 3600 (Malvern, UK), diluting samples in potassium nitrate (KNO_3_, 10^−2^M).

### 3.3. In Vitro Studies

#### 3.3.1. Cell lines and Culture Condition

The human breast adenocarcinoma cell line MDA-MB-231 was grown in DMEM-F12 (Lonza, Basel, Switzerland) supplemented with 10% FBS (Sigma, St. Louis, MO, USA), 1% non-essential amino acids (NEAA, Hyclone, South Logan, UT, USA), 1% sodium pyruvate 100 mM (Hyclone, South Logan, UT, USA), and 1% penicillin/streptomycin (Lonza, Basel, Switzerland).

Culture was maintained at 37 °C in a humidified atmosphere with 5% CO_2_. The cells were subcultured every 2 to 3 days by treatment with TrypLE™ Express Enzyme (Gibco) for MDA-MB-231.

#### 3.3.2. Nanodrug Preparation

Suspensions of the functionalized materials were prepared in culture media, without phenol red, at different concentrations depending on the experiments, always sonicating repeatedly before cellular incubation.

#### 3.3.3. Viability Assay

1 × 10^4^ cells were seeded per well in a 96-well culture plate for 48 h. After that, cells were incubated 24 h at 37 °C with dispersions of each MSN-functionalized material in culture medium without phenol red at different final tin concentrations (1 μM, 2.5 μM, 5 μM, 10 μM, and 25 μM). MSN bare material was incubated at the same silica concentration of the other organotin-bearing materials to evaluate the potential cytotoxicity of the material itself. After incubation, these solutions were discarded and replaced by 100 μL of medium, without phenol red and without serum, and 10 μL of a 12 mM MTT (dimethylthiazolyl-diphenyl-tetrazolium bromide) solution (in PBS 1X pH 7.4) was added to each well and mixed. After 3 h of incubation, all the supernatants less 25 μL were removed and 100 μL DMSO was added to each well to dissolve the formazan, leaving it 15 min to react. Cell viability was estimated by measuring absorbance at 570 nm using a *Unicam W-500 UV-Vis* plate reader. The absorbance values obtained were subtracted from the absorbance value of the “white wells” (only medium with MTT, no cells). The percentage of viability was calculated taking as 100% of viability the absorbance of the negative control wells (cells without material), and also positive control wells (cells incubated with DMSO) were studied.

#### 3.3.4. Cellular Uptake

Total of 2.5 × 10^4^ MDA-MB-231 cells were seeded in a 6-well culture plate and incubated overnight with the nanomaterial (MSN-AP-FA-PEP-S-Sn-AX) in a 1 μM final tin concentration. Then cells were fixed with 4% paraformaldehide (PFA) pH = 7.4 for 15 min at RT. After that, cells were washed three times with PBS 1X (5 min each) and permeabilized with 0.1% Triton X-100 in PBS for 5 min. Cells were rinsed three times with PBS 1X (5 min each) and then incubated for 20 min at RT with a blocking solution of 1% FBS in PBS 1X. Cells were rinsed again three times with PBS 1X (5 min each) and finally they were treated for 45 min at RT with a F-actin specific stain, AlexaFluor 488 Phalloidin (1:500 in PBS). Cells were washed with PBS and stained with DAPI (0.1% in PBS) for 5 min. The images were taken using a confocal microscope Zeiss LSM 780 (20×) under 37 °C and 5% CO_2_ and they were processed with ImageJ Fiji and Imaris software.

#### 3.3.5. Wound Healing Assay

1 × 10^5^ MDA-MB-231 cells were seeded per well in a 24-well culture plate for 48 h, until a cell monolayer was created. The monolayer was scratched with a pipette tip, the medium was absorbed with vacuum and the wells were cleaned twice with PBS without Ca or Mg. Then, the cells were incubated with the different nanomaterials at different concentrations (1 μM, 2.5 μM, 5 μM, and 10 μM) and the migration into the gap was imaged 0 and 24 h later using a Nikon Time Lapse microscope.

### 3.4. In Vivo Studies

#### 3.4.1. Animals and Ethics

Mice were housed in specific facilities (pathogen-free for mice) at the Spanish National Center for Cardiovascular Research (CNIC, Madrid, Spain). All animal experiments were carried out after previous approval by the ethics and animal welfare committee at CNIC and were in agreement with the Spanish Legislation and UE Directive 2010/63/EU.

#### 3.4.2. Breast Cancer Mouse Model

The breast cancer was generated by injecting subcutaneously 5 × 10^5^ MDA-MB-231 cells (in a 20 μL mixture of PBS 1X and Matrigel, 1.86:1 v/v) into 8 week old NOD Scid IL2 receptor gamma chain KO female mice, in their fourth left breast. Animals were housed at 22 °C, under a 12 h light/dark cycle with freely available water and food. The tumor was generally generated within the next 15–20 days after the injection.

#### 3.4.3. Treatment with Nanoparticles and in Vivo Fluorescence Imaging

Mice (*n* = 3/group) were injected i.v. into the tail vein every 2–3 days with 150 μL of the materials solutions at 420 μM over 10 days (5 injections). This therapeutic regimen was chosen based on our and others’ previous studies [50,52,75,76]. Two hours following the last injection, in vivo fluorescence imaging of Alexa Fluor 647 was performed on mice anesthetized with isoflurane gas. Murine models without nanoparticles treatment were used as control. Fluorescence in vivo imaging was performed with the IVIS Imaging System 200 series (Xenogen) (acquisition parameters: Cy5.5 ex/em filter, high level, BIN-HR, FOV: 6.6, f8). The region-of-interest (ROI) comparison was carried out by applying a constant ROI area to each image and processed using the Living Image software version 4.4 from Caliper Life Sciences. Fluorescence intensity was quantified as radiant efficiency accumulated over the 10 days treatment with MSN-based nanomaterials.

#### 3.4.4. Tumor Measurement

In order to evaluate the tumor growth, all mice were monitored by caliper measurements of tumor width (W) and length (L). Tumor volume was determined using the formula V = (L × (W^2^))/2 and volumes were normalized and compared with respect to the initial volumes.

#### 3.4.5. Statistic and Analysis

Data analysis was performed using the Prism 6 software (GraphPad, San Diego, CA, USA), and all chart data were expressed as mean ± standard deviation (Mean ± SD).

## 4. Conclusions

Triple negative breast cancer (TNBC) is the most aggressive subclass within human breast adenocarcinoma type and the development of a suitable therapeutic paradigm is still a great challenge. Toward this scope, in this work, we have successfully demonstrated that mesoporous silica nanoparticles are suitable nanoplatforms enabling the creation of a controlled delivery system of a metallodrug together with imaging abilities in order to promote the targeted theranosis of breast cancer. In more detail, a targeted nanodrug candidate functionalized with organotin(IV) complex (therapeutic), Alexa Fluor 647 NIR dye (diagnostic), and folate fragment (active targeting) moieties useful for the theranosis of human breast adenocarcinoma in mice models has been achieved. Furthermore, the application of an enzyme-responsive GFGL peptide linker for the site-selective controlled delivery of the metallodrug and triggered only after tumor cell uptake has been designed and successfully applied. Thanks to the combination of the receptor-mediated site targeting and the specific fine-tuned release mechanism, the MSN-AP-FA-PEP-S-Sn-AX (AX-3) nanotheranostic drug has showed enhanced therapeutic activity and reduced off-target toxicity after in vitro as well as in vivo applications in orthotopic mouse model of human TNBC. Considering the achieved results, the intrinsic flexibility of MSN materials and the possibility to couple other complementary therapeutic protocols, we are fully confident that our proposed strategy can pave the way toward the development of a more powerful generation of engineered nanotheranostics for the multitherapeutic treatment of triple negative breast cancers.

## Supplementary Materials

The following are available online at https://www.mdpi.com/2072-6694/12/1/187/s1, Figure S1: FT-IR spectrum of the functionalized materials MSN-AP and MSN-AP-FA-Sn, Figure S2. FT-IR spectrum of the functionalized materials MSN-AP-FA-PEP-S-Sn and AX-3, Figure S3. TG of the materials MSN, MSN-AP-Sn, MSN-AP-FA-Sn and MSN-AP-FA-PEP-S-Sn, Figure S4. 119Sn MAS NMR spectrum of MSN-AP-FA-PEP-S-Sn, Figure S5. 29Si MAS NMR spectrum of MSN-AP, Figure S6. 29Si MAS NMR spectrum of MSN-AP-FA-PEP, Figure S7. DR-UV spectra of the Alexa Fluor-functionalized materials MSN-AP-Sn-AX (AX-1), MSN-AP-FA-Sn-AX (AX-2) and MSN-AP-FA-PEP-S-Sn-AX (AX-3), Figure S8. Fluorescence imaging of MSN nanomaterials after coupling reaction with NIR dye Alexa Fluor 647, Figure S9. Tumor mass weight comparison after post mortem excision. Figure S10. Serum levels of alanine aminotransferase (ALT), aspartate aminotransferase (AST), alkaline phosphatase (AKP), albumin (ALB) and blood urea nitrogen (BUN) before (blue) and after (red) 10 days of different nanotherapies (n=3). Table S1. Mass loss (110 ºC-700 ºC) from thermogravimetry analyses, Table S2. Molar ratio S:Sn and Si:Sn obtained by XRF analyses., Table S3. Surface ζ-potential of the MSN nanomaterials.
