# Supplementary material for: Multifunctional Silica-Based Nanoparticles with Controlled Release of Organotin Metallodrug for Targeted Theranosis of Breast Cancer
Source: Cancers (Basel). 2020 Jan 12;12(1):187. doi: 10.3390/cancers12010187 (PMC7017138; doi:10.3390/cancers12010187)
Supplement: Supplementary file 1 [file cancers-12-00187-s001.pdf]

Multifunctional Silica-Based Nanoparticles with Controlled Release of Organotin Metallodrug for Targeted Theranosis of Breast Cancer.

Karina Ovejero Paredes,[1,2] Diana Díaz-García,[3] Victoria García-Almodóvar,[1,3] Marzia Marciello,[1] Laura Lozano Chamizo,[1] Miguel Díaz-Sánchez, [3] Sanjiv Prashar,[3] Santiago Gómez-Ruiz,[3*] and Marco Filice[1,2*]

[1] Nanobiotechnology for Life Sciences Group, Department of Chemistry in Pharmaceutical Sciences, Faculty of Pharmacy, Universidad Complutense de Madrid (UCM), Plaza Ramón y Cajal s/n, E-28040 Madrid, Spain; kovejero@ucm.es (K.O.P.); v.garciaalm@alumnos.urjc.es (V.G.-A.); laurloza@ucm.es (L.L.C.); marmarci@ucm.es (M.M.)

[2] Microscopy and Dynamic Imaging Unit, Fundación Centro Nacional de Investigaciones Cardiovasculares Carlos III (CNIC), Calle Melchor Fernandez Almagro 3, E-28029 Madrid, Spain

[3] COMET-NANO Group. Department of Biology and Geology, Physics and Inorganic Chemistry, ESCET, Universidad Rey Juan Carlos, Calle Tulipán s/n, E-28933 Móstoles (Madrid), Spain; diana.diaz@urjc.es (D.D.-G.); miguel.diaz@urjc.es (M.D.-S.); sanjiv.prashar@urjc.es (S.P.)

* Correspondence: santiago.gomez@urjc.es (S.G.-R.); mfilice@ucm.es (M.F.)

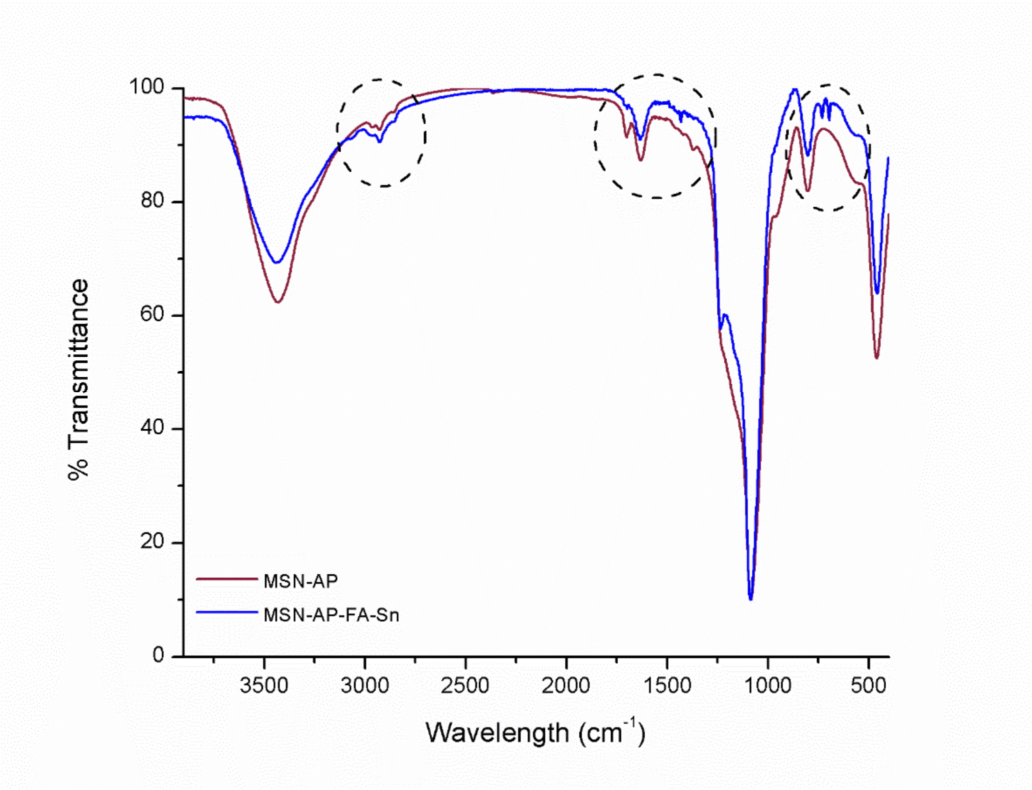

Figure S1. FT-IR spectrum of the functionalized materials MSN-AP and MSN-AP-FA-Sn.

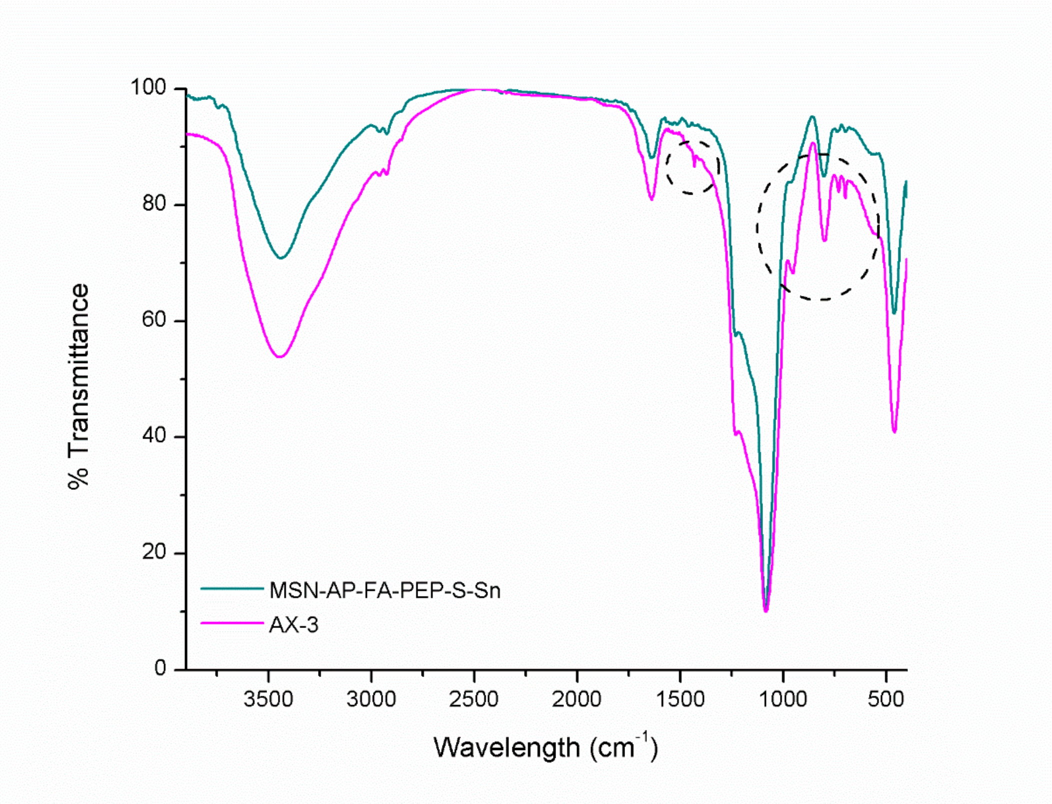

Figure S2. FT-IR spectrum of the functionalized materials MSN-AP-FA-PEP-S-Sn and AX-3.

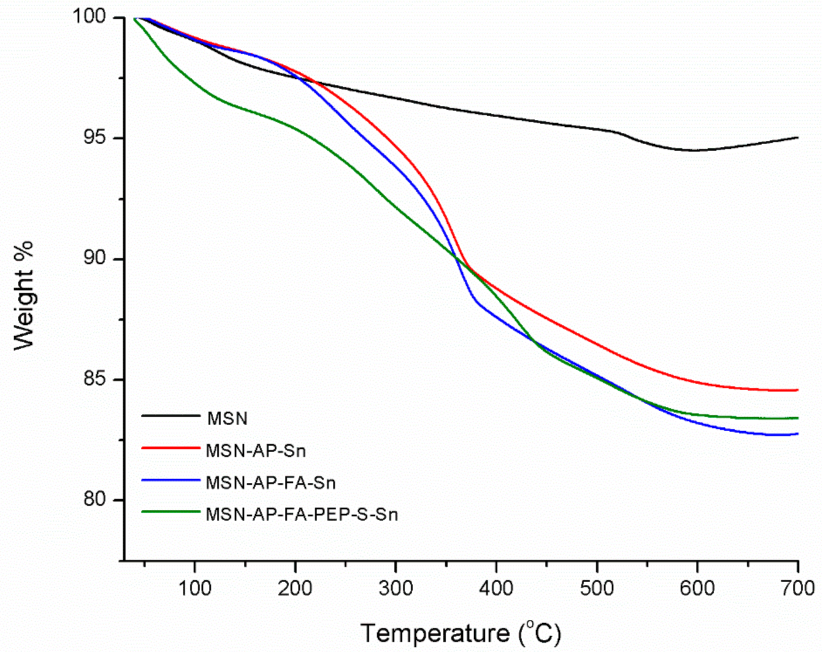

Figure S3. TG of the materials MSN, MSN-AP-Sn, MSN-AP-FA-Sn and MSN-AP-FA-PEP-S-Sn.

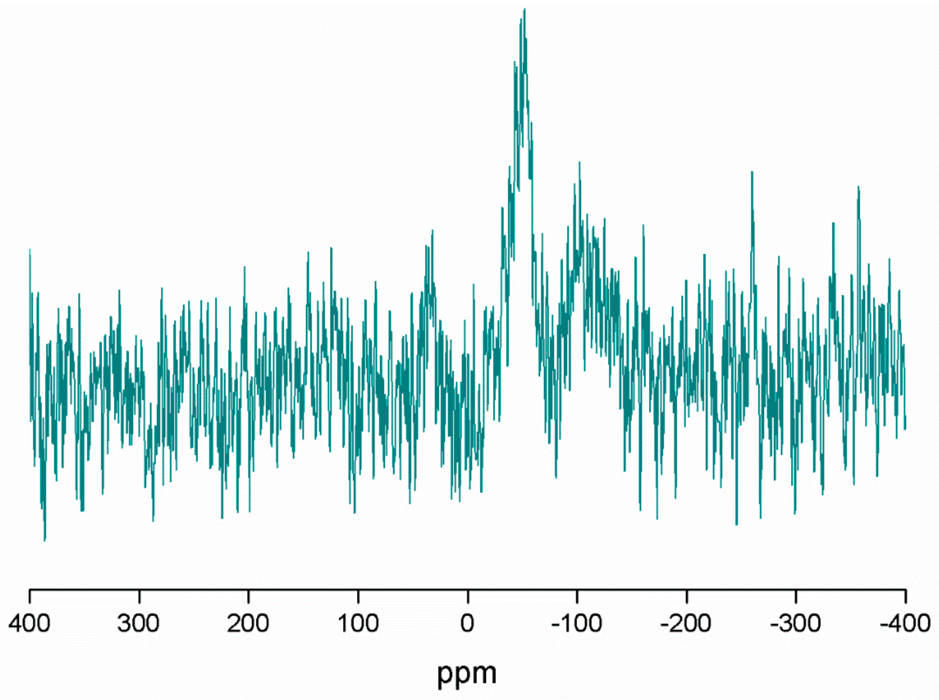

Figure S4. ^{119}Sn MAS NMR spectrum of MSN-AP-FA-PEP-S-Sn.

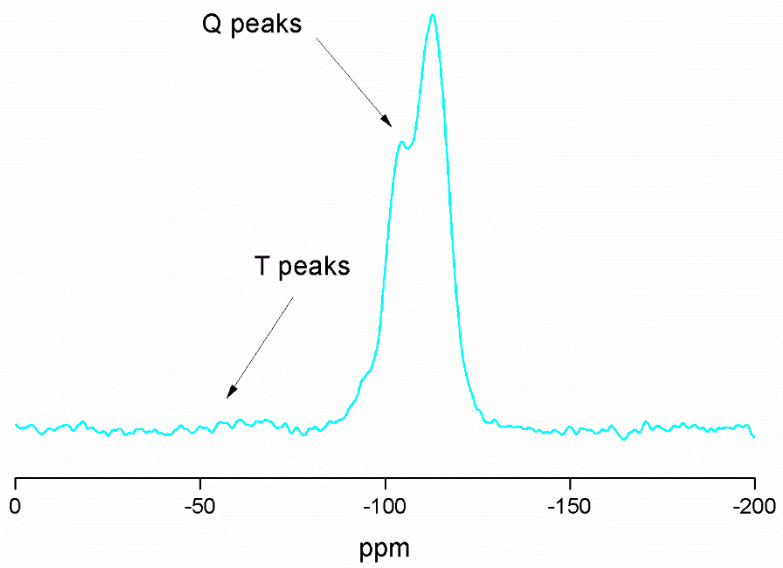

Figure S5. ^{29}Si MAS NMR spectrum of MSN-AP.

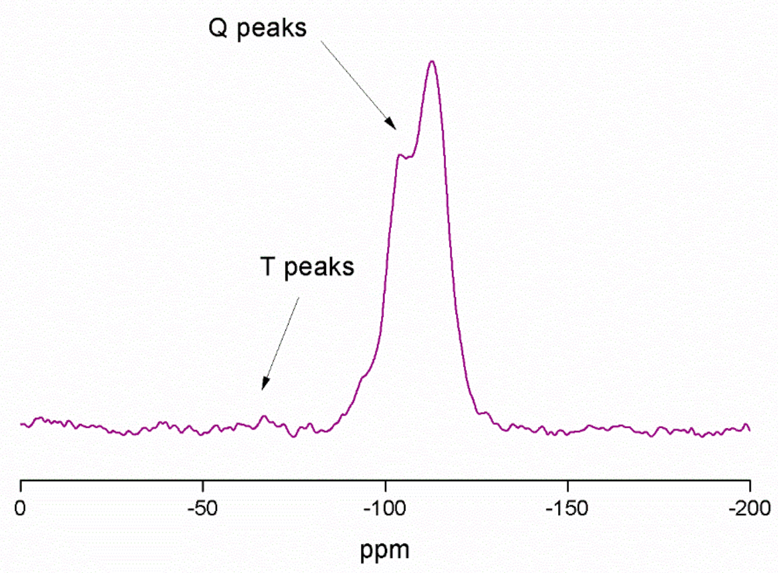

Figure S6. ^{29}Si MAS NMR spectrum of MSN-AP-FA-PEP.

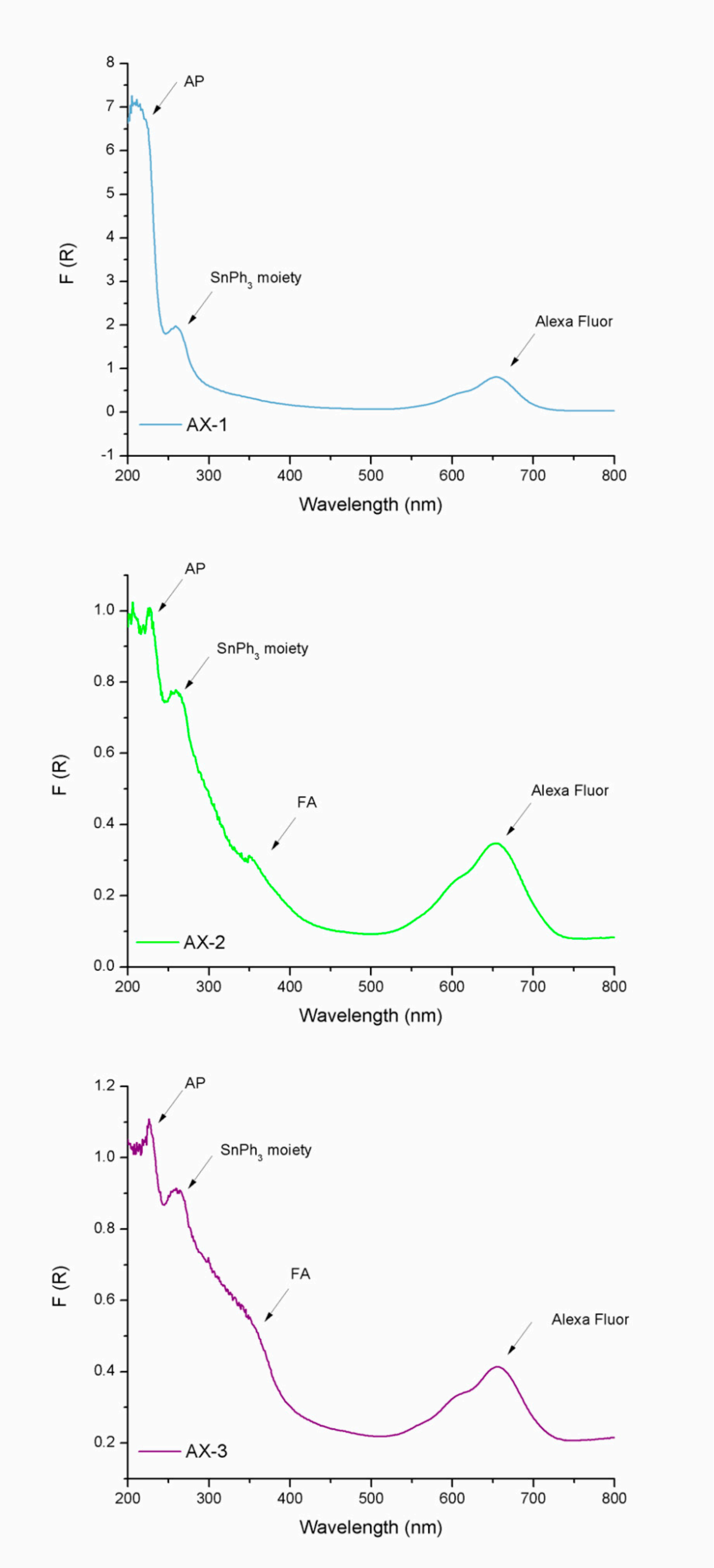

Figure S7. DR-UV spectra of the Alexa Fluor-functionalized materials MSN-AP-Sn-AX (AX-1), MSN-AP-FA-Sn-AX (AX-2) and MSN-AP-FA-PEP-S-Sn-AX (AX-3).

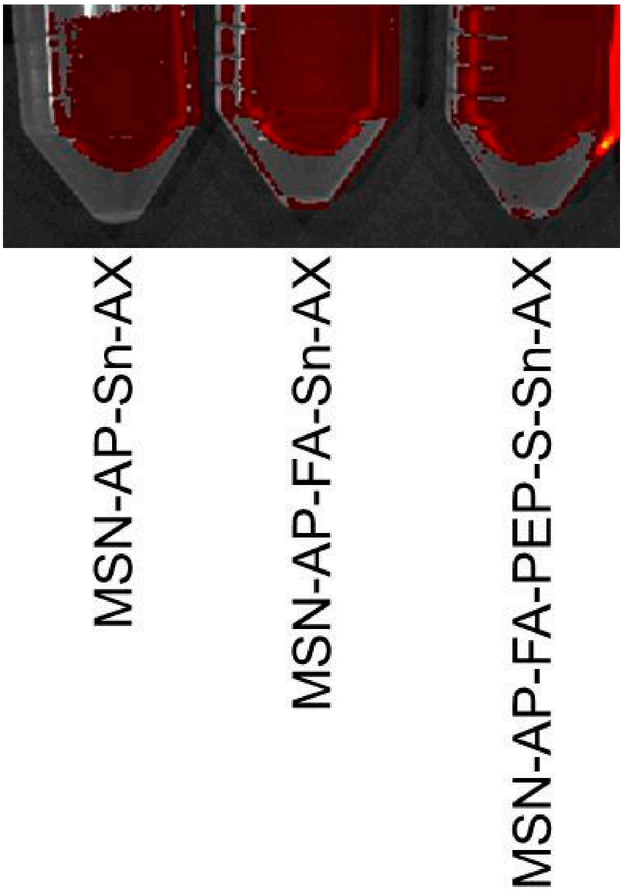

Figure S8. Fluorescence imaging of MSN nanomaterials after coupling reaction with NIR dye Alexa Fluor 647.

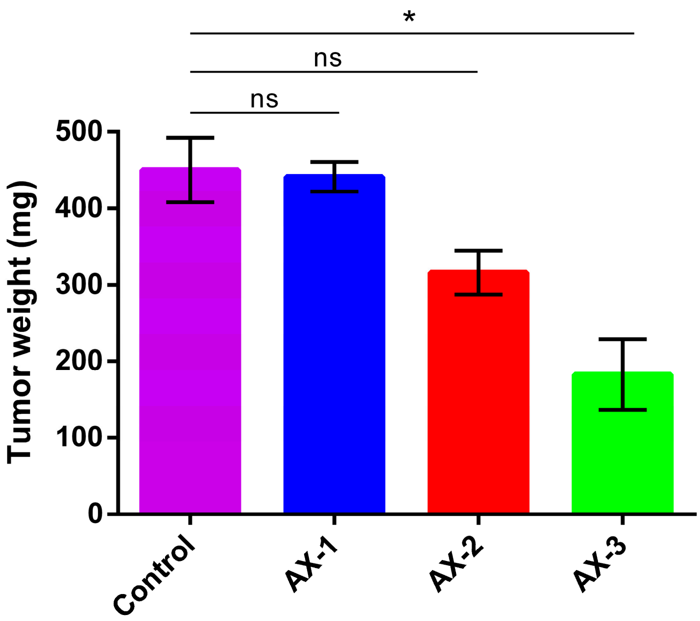

Figure S9. Tumor mass weight comparison after post mortem excision. Significance was calculated by unpaired t-test of One-way ANOVA. *ns*: $p > 0.05$ or not significant statistical difference between the groups of data; *: $p < 0.05$ or significant statistical difference between the two groups of data

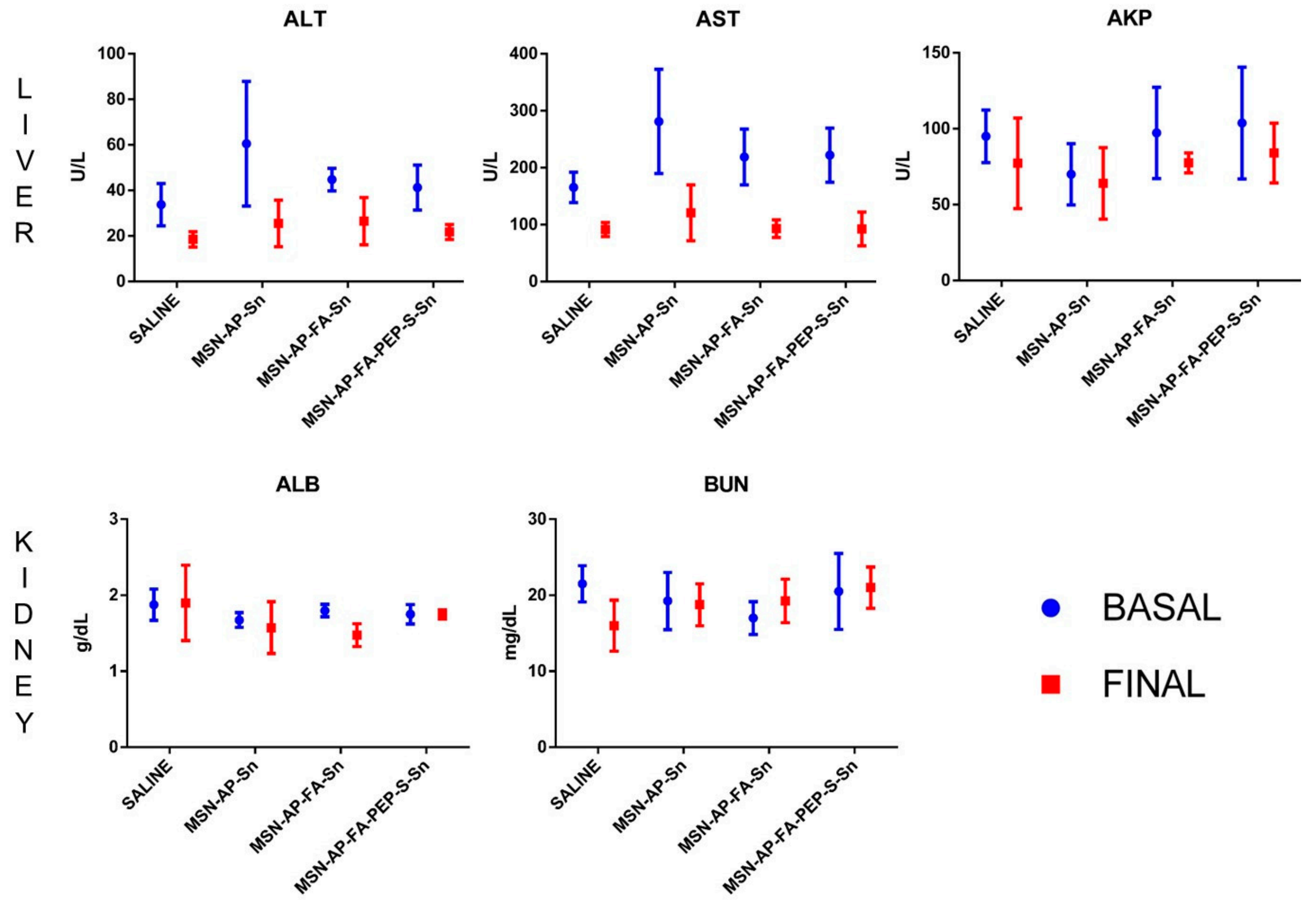

Figure S10. Serum levels of alanine aminotransferase (ALT), aspartate aminotransferase (AST), alkaline phosphatase (AKP), albumin (ALB) and blood urea nitrogen (BUN) before (blue) and after (red) 10 days of different nanotherapies (n=3). By applying unpaired t-test of One-way ANOVA analysis, not significant statistical differences were identified in all cases ($p > 0.05$).

Supporting Tables

Table S1. Mass loss (110 ºC-700 ºC) from thermogravimetry analyses.

MATERIAL	Mass loss (%)
**MSN**	3.82
**MSN-AP-Sn**	14.44
**MSN-AP-FA-Sn**	16.19
**MSN-AP-FA-PEP-S-Sn**	13.57

Table S2. Molar ratio S:Sn and Si:Sn obtained by XRF analyses.

MATERIAL	S:Sn	Si:Sn
**MSN-AP-Sn**	1:5.04	1:0.24
**MSN-AP-FA-Sn**	1:4.57	1:0.24
**MSN-AP-FA-PEP-S-Sn**	1:2.70	1:0.08

Table S3. Surface ζ-potential of the MSN nanomaterials.

MATERIAL	pI(pH)
**MSN**	3.5
**MSN-AP**	7.3
**MSN-AP-Sn**	6.2
**MSN-AP-FA-Sn**	5.1
**MSN-AP-FA-PEP-S-Sn**	6.4
